# Genome-Wide Identification, Characterization, Expression Analysis, and Interacting Protein Prediction of the *GSK3/Shaggy-like* Gene Family in Watermelon

**DOI:** 10.3390/plants15030484

**Published:** 2026-02-04

**Authors:** Peng Tian, Jingjing Zhang, Bowen Liu, Xiurui Gao, Bing Li, Wei Liu, Yanrong Wu

**Affiliations:** Institute of Cash Crops, Hebei Academy of Agriculture and Forestry Sciences, Shijiazhuang 050051, China; tianpengtdc@163.com (P.T.); 1109jing@163.com (J.Z.); bwl537003@gmail.com (B.L.); shijiagao73@126.com (X.G.); lbhbnky@163.com (B.L.); liuwei44856@sina.com (W.L.)

**Keywords:** watermelon, GSK3, brassinosteroid, stress-responsive

## Abstract

Glycogen synthase kinase 3 (GSK3/Shaggy-like) is a highly conserved serine/threonine kinase that orchestrates growth, hormone signaling, and abiotic stress responses in both animals and plants, yet its role in watermelon remains unexplored. In this study, we conducted a whole-genome identification, identifying a total of eight members of the *GSK3* gene family (*ClGSK3*) distributed across seven chromosomes. Phylogenetic and synteny analyses resolved the eight *ClGSK3s* into four subfamilies that display one-to-one or one-to-many orthology with *Arabidopsis* and rice *GSK3* genes, indicating conserved genomic micro-collinearity across dicots and monocots. Predictions of cis-acting elements and transcriptome data analysis indicate that *ClGSK3s* may be involved in hormone- and stress-responsive conditions. Protein–protein interaction networks predicted 53 candidate partners for five ClGSK3 proteins; yeast two-hybrid assays subsequently confirmed that ClSK21 associates with three of them—orthologs of the core brassinosteroid (BR)-signaling components BKI1 and BZR1. qRT-PCR revealed that *ClSK21*, *ClSK31*, and *ClSK41* are rapidly and significantly reprogrammed by BR treatment. Collectively, our data suggest that *ClGSK3s* modulate fruit development and stress tolerance by integrating hormone-related pathways, especially BR signaling. Future studies are encouraged to integrate genetics and multi-omics approaches to systematically validate the roles of *ClGSK3s* in hormone signaling and abiotic stress responses.

## 1. Introduction

Watermelon (*Citrullus lanatus*, 2n = 2× = 22), a member of the Citrullus genus in the Cucurbitaceae family, originated in Africa [1]. It is highly valued for its nutritional content and refreshing properties, making it a significant vegetable crop globally [2,3]. China has long been the leading producer and consumer of watermelon worldwide, with projections indicating its continued dominance in 2023 [4]. The focus of breeding efforts has shifted towards developing varieties with high yield, quality, and resistance [5]. Nevertheless, the molecular mechanisms governing watermelon growth, development, and stress responses are not well elucidated compared to other major crops [6,7].

Glycogen synthase kinase 3 (GSK3)/Shaggy-like kinases are a highly conserved family of serine/threonine protein kinases essential for various cellular and developmental processes in eukaryotes. In animals, GSK3 proteins are involved in signal transduction pathways regulating glycogen synthesis, insulin activity, transcriptional activation, and development [8]. Similarly, in plants, the *GSK3* gene family plays a critical role in physiological responses like osmotic stress, wound healing, and Oleandrin lactone signaling [9]. The first plant *GSK3* gene was identified in *Medicago sativa*. With the advancement of genome sequencing in various plant species, comprehensive studies on the identification and functional analysis of *GSK3* gene families have been carried out in crops such as wheat, *Nicotiana tabacum* and pear [10,11,12]. The *GSK3* genes are typically categorized into four subfamilies, mirroring the classification of their homologous genes [13,14].

The *GSK3* genes have been extensively studied in model species such as *Arabidopsis* and rice. They are implicated in hormone signaling and environmental adaptation. For instance, in *Arabidopsis*, BIN2 (AtSK21), a well-studied GSK3 family member, phosphorylates various substrates like EGL3, TTG1, BZR1, and SnRK2.2, influencing root hair formation, Brassinosteroid (BR) and Abscisic Acid (ABA) signaling pathways, among others [15,16,17,18,19,20,21]. AtSK11, AtSK22, and AtSK31 are known to enhance plant salt tolerance [19,22,23]. In rice, OsSK21 is highly expressed in the panicle and plays a crucial role in determining rice plant morphology, panicle architecture, and resistance to abiotic stress [24,25]. OsSK23 functions as a negative regulator of BR signaling, acting synergistically with factors such as OsPPKL1 and OsBSK3 [26,27]. OsSK22 serves as the central kinase in BR signaling, regulating traits such as plant architecture, panicle shape, and disease resistance through the phosphorylation of various downstream factors [28,29,30,31]. Additionally, it interacts with pathways involving jasmonic acid, strigolactone, and others [32,33,34]. OsSK41 regulates rice grain shape and weight via the auxin pathway and is associated with salt stress resistance [35,36,37,38]. Despite their evolutionary conservation, the biological roles of *GSK3* genes can vary among species and contexts. To date, no comprehensive study has explored the *GSK3* gene family in watermelon, limiting our understanding of its potential roles in fruit development and abiotic stress tolerance.

Given the crucial functions of *GSK3* genes, we hypothesize that the *GSK3* gene family in watermelon is evolutionarily conserved and plays analogous roles in mediating fruit development, plant architecture, and tolerance to abiotic stresses by integrating hormone signaling pathways, particularly BR signaling. The aim of this study is to conduct a genome-wide identification and systematic characterization of the *ClGSK3* gene family. By achieving this aim, we seek to provide the first comprehensive overview of the *ClGSK3* family, clarify their potential functional roles in watermelon, and identify key molecular targets that can be utilized in breeding programs to develop climate-resilient and high-quality watermelon varieties. This work lays a foundation for further functional validation of *ClGSK3* genes and their practical application in watermelon genetic improvement.

## 2. Results

### 2.1. Identification of GSK3/Shaggy-like Family Members in Watermelon

To identify GSK3/Shaggy-like family genes in watermelon, we conducted BLAST (https://blast.ncbi.nlm.nih.gov/, accessed on 3 November 2025) searches using the protein sequences of all 10 members from *Arabidopsis* and 9 members from rice as queries. To ensure the reliability of the BLAST results, we obtained the kinase domain model Pkinase (PF00069.23) and the hidden Markov model (HMM) of AtSKs protein kinase from the Pfam website. We then employed these models to further search for and confirm the members of the GSK3 family in watermelon. We identified a total of eight members, which we designated as ClSK11, ClSK12, ClSK13, ClSK21, ClSK22, ClSK23, ClSK31, and ClSK41, based on their closest phylogenetic relationships with *Arabidopsis* and the rice family (Table S1).

Further analysis of GSK3 proteins entailed evaluating their physical and chemical properties. Predicted amino acid counts varied from 379 (ClSK21) to 469 (ClSK31), corresponding to molecular weights ranging from 43.03 kDa (ClSK21) to 52.75 kDa (ClSK31) (Table S2). The predicted isoelectric point (pI) suggested that, apart from ClSK31 (pI = 6.61), all family members were expected to be basic proteins with pI values above 7. Moreover, all the 8 proteins displayed an average grand average of hydropathy (GRAVY) score below 0, indicating their hydrophilic nature. Examination of the instability index indicated that ClSK21, ClSK22, and ClSK23 proteins were anticipated to be unstable, with instability index values exceeding 40 (Table S2).

### 2.2. Structural and Phylogenetic Analysis

**Figure 1:**
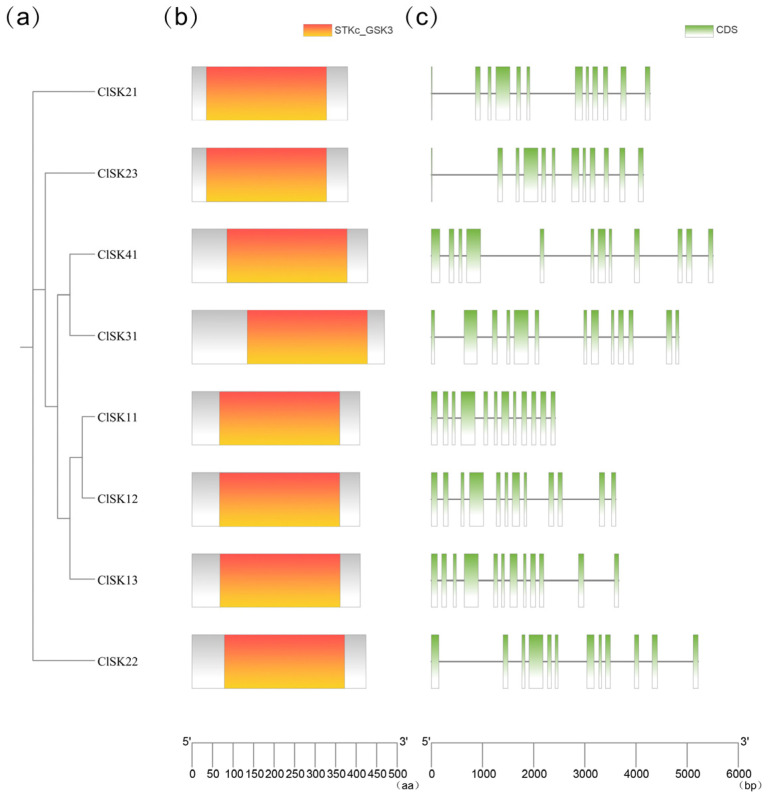
Phylogenetic relationships, conserved domains and gene structures of *Glycogen synthase kinase 3 (GSK3)* genes in watermelon: (**a**) Phylogenetic relationships of ClGSK3 proteins. (**b**) Conserved domains in ClGSK3 proteins (**c**) Display of intron/exon structures of *ClGSK3* genes.

To delve into the functional differentiation and evolutionary path of the watermelon *GSK3* gene family, we initially generated a maximum likelihood (ML) phylogenetic tree using IQ-TREE software (v2.4.0) based on full-length amino acid sequences (Figure 1a). Subsequently, we delineated the conserved protein domains (Figure 1b) and the exon-intron architecture of the genes (Figure 1c).

**Figure 2:**
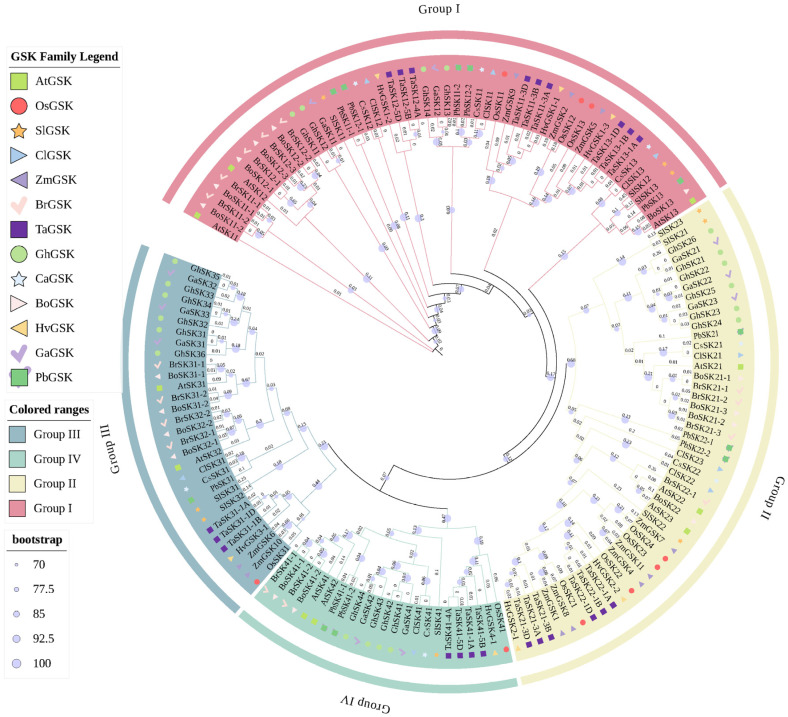
The phylogenetic tree of *GSK3* gene family in 13 representative plant species. The maximum likelihood (ML) method and 1000 bootstrap replicates were used to construct phylogenetic tree containing 154 GSK3 proteins. Bootstrap values and branch length are shown at the nodes. Proteins are prefixed by species abbreviations: At, *Arabidopsis thaliana*; Os, *Oryza sativa*; Zm, *Zea mays*; Ta, *Triticum aestivum*; Br, *Brassica rapa*; Bo, *Brassica oleracea*; Ga, *Gossypium arboreum*; Gh, *Gossypium hirsutum*; Cs, *Cucumis sativus*; Hv, *Hordeum vulgare*; Pb, *Pyrus bretschneideri*; Sl, *Solanum lycopersicum*; Cl, *Solanum lycopersicum*. The four major clades (Groups I–IV) are indicated by colored brackets on the left.

Subsequently, an ML phylogenetic tree encompassing 154 GSK3 proteins from 13 species, including *Arabidopsis*, rice, wheat and maize (Table S2), was constructed using full-length sequences in IQ-TREE software. The analysis unveiled four distinct clusters (I–IV), each containing members from the three species, indicating an early differentiation before speciation. Notably, Group I included ClSK11/12/13, Group II contained ClSK21/22/23, Group III had ClSK31, and Group IV included ClSK41 (Figure 2).

Our findings indicated that the phylogenetic distinct clusters of *ClGSK3s* correlate with both protein structure and gene structure. At the protein level, while each member contains a single characteristic Pkinase domain, the domain composition and length among members of each group are highly consistent (Figure 1b, Figure 2 and Figure S1). At the gene level, the number of exons ranged from 11 to 13: Group I genes had 12 exons, Group II had 11 exons (except for *ClSK22*), with substantial diversity in intron phase and length, which might reflect ancestral indel events or possible transcript isoforms. This “phylogenetic-structural” association pattern implies that post-gene duplication, structural reconfiguration, and functional diversification may follow a progressive trajectory of “initial conservation followed by subsequent diversification.” These insights offer pivotal clues for future functional validation and molecular engineering endeavors.

### 2.3. Chromosomal Localization and Collinearity Analysis

**Figure 3:**
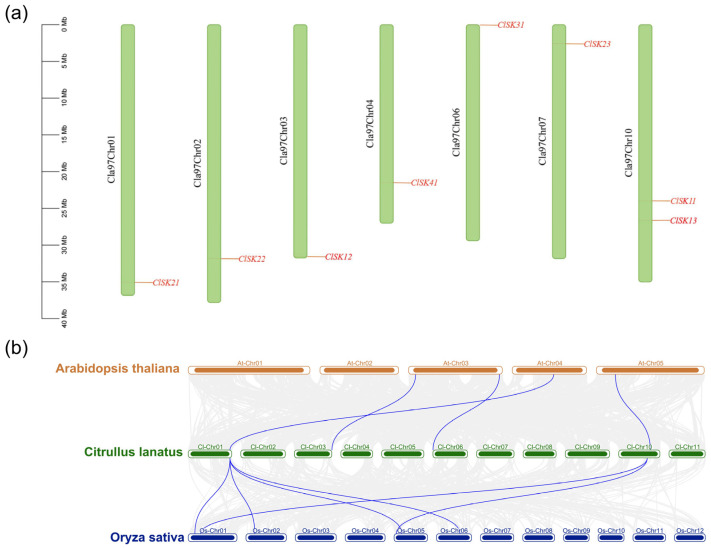
The distribution of *GSK3* gene family members of chromosomes in *C. lanatus* and collinearity analysis of *GSK3* gene family in *A. thaliana*, *C. Lanatus* and *O. Sativa*: (**a**) The distribution of *GSK3* gene family members of chromosomes in *C. lanatus*; (**b**) Collinearity analysis of *GSK3* gene family in *A. thaliana*, *C. Lanatus* and *O. Sativa*. The red lines delineate the syntenic *GSK3* gene pairs.

To ascertain the chromosomal localization of *GSK3* genes in watermelon, we conducted an analysis of their physical distribution across chromosomes. Among the 8 *GSK3* genes, they are distributed evenly across 7 of the 11 watermelon chromosomes. Chromosome 10 contains 2 genes, while chromosomes 1, 2, 3, 4, 6, and 7 each harbor 1 gene (Figure 3a).

Gene duplication, including whole-genome duplication (WGD), segment duplication, and tandem duplication, is widespread in plant genomes and is a significant factor in genome evolution, resulting in the substantial enlargement of plant gene families [39]. A collinearity analysis of *GSK3* genes in *Arabidopsis*, rice, and watermelon was performed to clarify the evolutionary mechanism of the *GSK3* family. The analysis identified 4 *GSK3* genes in rice, 4 in watermelon, and 6 homologous genes in *Arabidopsis*, resulting in the formation of 10 collinear pairs (Figure 3b, Table S3), which suggests a shared ancestry among these genes.

The synonymous substitution rate (Ks) of all colinear gene pairs was estimated with the MCScanX module implemented in TBtools. Among the *GSK3* pairs, the four *Arabidopsis*–watermelon colinear fragments exhibited Ks < 1, whereas the six watermelon–rice fragments yielded Ks > 1 (Table S3). This distribution indicates a closer evolutionary distance between watermelon and the dicot model *Arabidopsis* than between watermelon and the monocot rice, consistent with the divergence of dicotyledonous and monocotyledonous lineages. Importantly, all colinear *GSK3* gene pairs displayed Ka/Ks ratios significantly < 1, reflecting strong purifying selection that has acted to conserve *GSK3* function across these taxa.

The Orthogroup assignment was derived from OrthoFinder (v2.5, Emms DM et al., United Kingdom) default settings, grouping the 10 collinear genes into 3 orthogroups (Table S4). These 10 pairs show a mix of one-to-one and one-to-many orthology (Table S4), suggesting ancient duplications within the watermelon lineage after the monocot–dicot split.

### 2.4. Promoter Cis-Regulatory Elements Analysis

**Figure 4:**
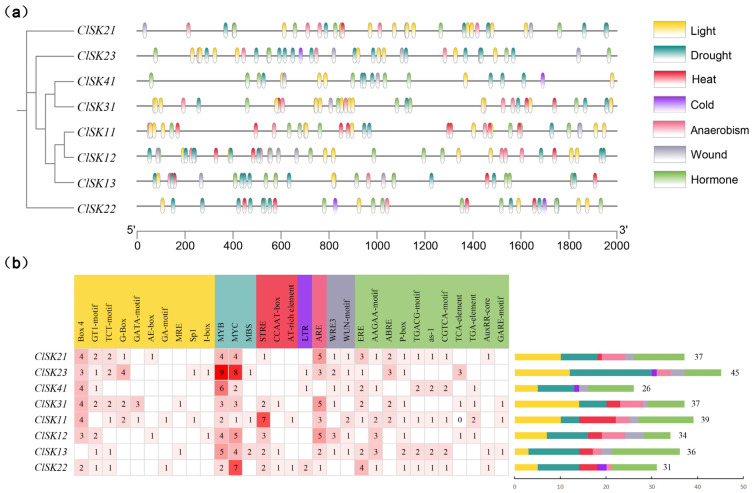
Identification of cis-acting elements of *GSK3* genes in watermelon: (**a**) The cis-acting elements distribution in the *GSK3* gene promoter of watermelon. The cis-acting elements are categorized into seven groups: hormone, light, anaerobism, drought, heat, wound and cold. (**b**) The left side illustrates the functions of cis-acting elements along with their corresponding numbers. The quantity of each type of cis-acting element within each promoter sequence is represented on the right side by distinct colored bars, which correspond to the groups identified in (**a**). Each row represents a gene, which is identified on the leftmost side.

Regulation of plant gene transcription relies on controlling cis-regulatory elements (CREs) within promoter regions, influencing gene expression in response to environmental stresses and tissue-specific contexts. To explore the transcriptional regulatory network of the *GSK3* gene family in watermelon, we analyzed the 2000 bp upstream promoter sequence and predicted CREs using the PlantCARE tool. Our analysis identified enriched core elements related to hormone, light, anaerobism, drought, heat, wound, and cold signaling pathways in the promoter region of the *GSK3* gene family (Figure 4, Table S5), suggesting a key role in integrating light signals, hormone cues, and stress responses.

Group I (*ClSK11/12/13*) encompasses the most diverse array of elements, comprising 55 types, and includes numerous photoresponsive elements and stress-related elements. This suggests a robust capacity for stress adaptation and potential involvement in light-dependent processes, such as photomorphogenesis and the regulation of photosynthesis in plants. Group II (*ClSK21/22/23*) exhibited a high percentage of abundant hormone-responsive elements (35.2%) and photoresponsive elements (27.8%). These elements potentially play a role in light-dependent mechanisms, such as regulating flowering time. Group III (*ClSK31*) comprises an equal proportion of photoresponsive and hormone-responsive elements, each accounting for 33.3%. This group contains fewer element types, totaling 18, and does not include complex stress-responsive elements. It may play a role in regulating specific tissues or developmental stages, as well as in hormone regulation under particular photoperiods. Group IV (*ClSK41*) has the highest proportion of hormone-responsive elements (46.2%), includes unique cold-responsive elements (LTR), and possesses the smallest total number of elements (26). This group may play a crucial role in sustaining growth in low-temperature environments.

### 2.5. Tissue-Specific Expression of ClGSK3s in Watermelon

**Figure 5:**
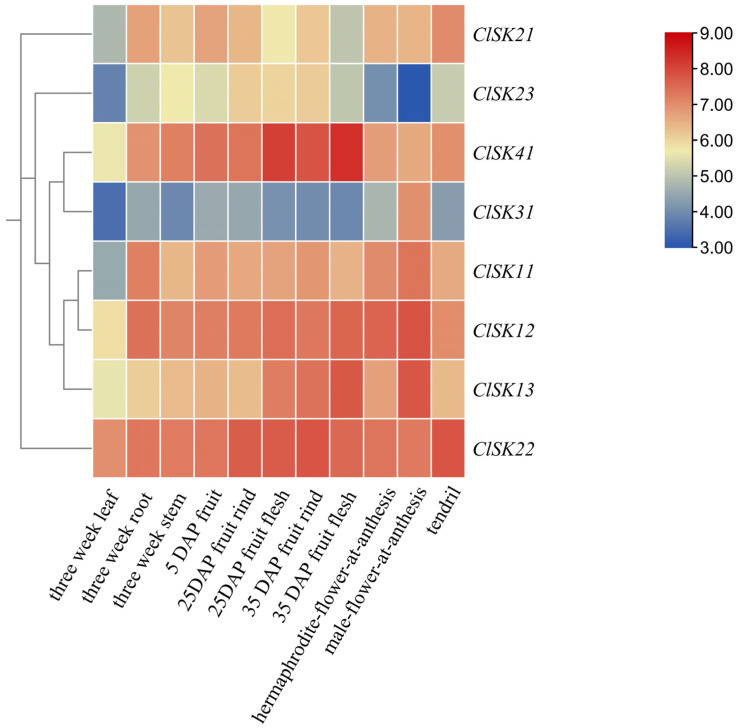
Tissue-specific expression profile of *ClGSK3s* genes. Expression levels of *ClGSK3* gene transcripts across various tissues are presented. DAP refers to days after pollination. The color scale indicates fold changes normalized by log2-transformed data, with blue signifying downregulated genes and red indicating upregulated genes.

The temporal and spatial expression of genes is closely linked to the manifestation of biological functions. In this study, *ClGSK3* genes were analyzed using the transcriptome database (Accession no. PRJNA676179) of watermelon across various tissues and developmental stages, including vegetative tissues (3-week-old leaves, roots, and stems), flower organs (bisexual and male flowers at anthesis), and fruit developmental series from 5 to 35 days after pollination [40]. The analysis revealed a generally consistent expression pattern across different tissues (Figure 5, Table S6). *ClSK41* exhibited a pronounced specificity for fruit, particularly in mature pulp, suggesting its potential involvement in glucose metabolism and accumulation. *ClSK23* demonstrated high expression during the mid-stage of fruit development, indicating a possible role in development and sugar transport. *ClSK22/21* showed elevated expression in the pericarp and tendrils, which may relate to plant morphology, fruit shape, and stress responses. *ClSK12/13/31* were predominantly expressed in floral organs, potentially linked to pollen development or sex determination. *ClSK11* was highly expressed in both flowers and roots, suggesting its involvement in basal energy metabolism.

We re-analyzed the published RNA-seq dataset (SRP143549) of four-leaf-stage drought-tolerant M20 and susceptible Y34 watermelon seedlings under 4 and 8 days of water-withholding (drought) treatment. Relative to fully irrigated controls, *ClSK12* and *ClSK41* were markedly up-regulated in both genotypes at both time points, pointing to a conserved, early role in drought perception. By contrast, *ClSK11* and *ClSK21* exhibited pronounced up-regulation only at 8 d, while *ClSK13* was significantly down-regulated at the same stage in both genotypes (Figure S2, Table S7), implying that these three genes participate in later-acting drought responses. *ClSK31* was constitutively higher in M20, suggesting a basal role in drought resilience. Then we reanalyzed the published RNA-seq dataset (GSE146087) obtained from six-week-old seedlings of the Crimson Sweet variety exposed to 300 mM NaCl [41]. Compared to deionized-water controls, all *ClGSK3s*, except for *ClSK31*, exhibited significant differential expression (Figure S3, Table S8), indicating their involvement in the salt-stress response.

### 2.6. Prediction and Validation of the Interacting Proteins of the ClGSK3s

**Figure 6:**
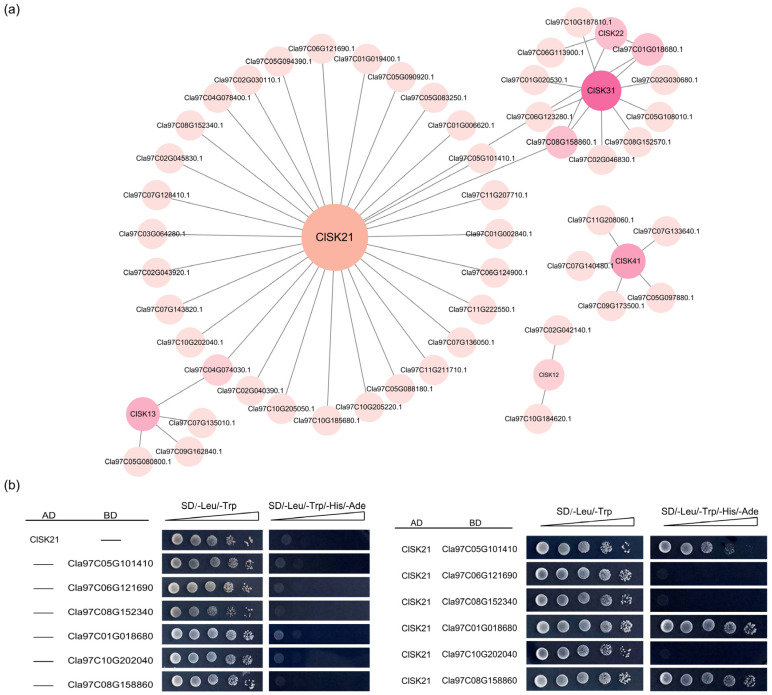
Predicted Protein–protein interactions (PPI) networks and interactions validation of ClGSK3s: (**a**) PPI networks of 5 ClGSK3s and predicted interaction proteins. Nodes represent watermelon proteins (97103_v2 Genome), with edges denoting interactions between them. The 5 ClGSK3s are highlighted in bold to emphasize their central hub positions within the network. (**b**) GAL4-based Y2H assay validation of the interaction of ClSK21 and 6 predicted interaction proteins. AD, activation domain; BD, binding domain; SD, synthetic dropout; gradients indicate tenfold serial dilutions.

Protein–protein interactions (PPIs) involving GSK3 proteins were examined to elucidate potential functions, signaling pathways, and metabolic processes. The STRING database facilitated the prediction of interactions among the eight ClGSK3 proteins, utilizing ortholog information primarily derived from *Arabidopsis*. A total of 53 potential interacting partners with five ClGSK3 proteins were identified (Figure 6a, Table S9). Among them, ClSK21 was predicted to interact with 23 proteins, ClSK31 with 9 proteins, ClSK41 with 5 proteins, ClSK13 with 4 proteins, ClSK22 with 3 proteins, and ClSK12 with 2 proteins (Figure 6a). KEGG analysis of these interacting proteins revealed their involvement in multiple metabolic pathways, including ribosome biogenesis and circadian rhythm, with the plant hormone signaling pathway containing the largest number of members (Table S9). This finding suggests a close relationship between the ClGSK3 protein and plant hormone signaling, particularly in the case of ClSK21, which has 16 of its 23 predicted interacting proteins associated with this pathway.

Only ClSK21 exhibited high-confidence interactions (Required score > 0.900) with six proteins: Cla97C05G101410, Cla97C06G121690, Cla97C08G152340, Cla97C01G018680, Cla97C10G202040, and Cla97C08G158860 (Table S9). These high-confidence interactions were validated through yeast two-hybrid experiments. The results indicate that ClSK21 has genuine interactions with Cla97C05G101410, Cla97C01G018680, and Cla97C08G158860 (Figure 6b). These three genes encode orthologs of the key BR signaling factors BKI1 (Cla97C05G101410) and BZR1 (Cla97C01G018680 and Cla97C08G158860) in watermelon. These findings suggest that *ClSK21* may play a regulatory role in BR signaling within this species. Further research is necessary to provide additional evidence.

### 2.7. Expression Pattern of GSK3s Response to BR Treatment

**Figure 7:**
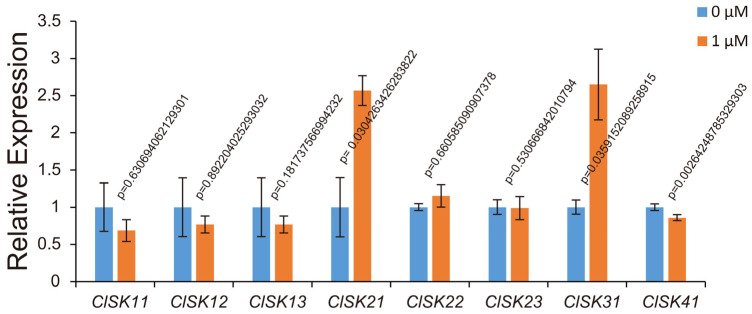
qRT–PCR analysis of *ClGSK3s* transcript levels in watermelon seedlings treated with exogenous BR. RNA samples were extracted from 7-day-old seedlings. Different colors indicate varying concentrations of BR treatment. Expression values were normalized to 0 µM exogenous BR treatment, processed relative values and are presented as means ± SD (*n* = 3). *p*-values from Student’s *t*-test. Measurements were conducted blind to individual seedling identity.

Promoter profiling, spatiotemporal expression atlases and predicted interactors all pointed to a BR link for *ClGSK3s*. To test this, we monitored the transcript abundance of all eight family members after 0 and 1 µM exogenous brassinolide (BL), a type of BR, treatment. Within the same time window, *ClSK21* and *ClSK31* transcripts rose 2.57- and 2.65-fold, respectively (*p* < 0.05), whereas *ClSK41* dropped to 85.8% of the control level (*p* < 0.01) (Figure 7). These reciprocal responses implicate *ClSK21*, *ClSK31* and *ClSK41*, which may participate in BR signaling.

## 3. Discussion

Glycogen synthase kinase 3/Shaggy-like kinases are highly conserved serine/threonine kinases that regulate plant growth, development, and stress responses through the integration of hormone signaling [10,11,12]. Prior to this study, the *GSK3* gene family in watermelon, an economically significant horticultural crop, had not been characterized. In this research, we systematically analyzed the identification, phylogeny, chromosomal distribution, cis-regulatory elements, transcriptome data, protein–protein interactions, and BR responsiveness of *ClGSK3s*. This work provides the first comprehensive insight into their potential functions and evolutionary dynamics.

Watermelon possesses only eight *GSK3* genes, fewer than most model plants and crops reported to date [10,42,43,44,45,46,47]. Nevertheless, all four canonical sub-clades (I–IV) are represented, suggesting a “minimal but complete” *GSK3* toolkit shaped by dicot-specific duplication and strong purifying selection. The evolutionary trajectory of the *GSK3* family was systematically examined by identifying all family members in 13 species (Table S2), as documented in previous studies [10,42,43,44,45,46,47]. Phylogenetic clustering of *ClGSK3s* into four subfamilies, each encompassing members from all examined species (Figure 2), indicates that the major lineages diverged prior to the monocot–dicot split and have since maintained structural conservation. Synteny and Ks analyses identify ten collinear ClGSK3–AtGSK3/OsGSK3 pairs (Table S3); dicot–dicot blocks exhibit Ks < 1, while dicot–monocot blocks present Ks > 1, thereby calibrating the evolutionary timeline of Cucurbitales. All collinear pairs exhibit Ka/Ks ≪ 1 (Table S3), indicating persistent purifying selection that maintains core kinase function. One-to-one and one-to-many orthologies trace the modern *ClGSK3s* complement to (i) ancient pre-eudicot duplications shared with *Arabidopsis* and (ii) a watermelon-specific tandem duplication event on chromosome 10, generating two paralogs (*ClSK11* and *ClSK13*). This duplication is absent in *Arabidopsis* and rice, indicating it occurred after the Cucurbitaceae lineage diverged from other dicots. Each ClGSK3 contains the Pkinase domain signature, and closely related genes exhibit similar exon–intron architecture and domain layout (Figure 1). Subfamily members share highly consistent domain composition and length at the protein level. At the gene level, exons vary from 11 to 13 (Group I comprises 12 exons, while Group II mostly comprises 11 exons except *ClSK22*). The diversity in intron phase and length may indicate ancestral indel events or the presence of transcript isoforms, underscoring a “phylogenetic–structural” correlation in which post-duplication rearrangements facilitate functional differentiation. Most proteins are basic and hydrophilic, making them suitable for cytoplasmic signaling, whereas *ClSK21/22/23* show high instability indices (>40) (Table S2), facilitating rapid hormonal or stress responses through conformational switching or targeted degradation.

Watermelon is distinguished by its high sugar content and large fruit size [2,48,49], and our data identify *ClGSK3s* as key regulators of these species-specific traits. Tissue profiles and promoter dissection converge to support a model in which *ClGSK3s* connect hormonal signals to developmental outcomes and fruit quality, with distinct functional specializations aligned to their subfamily-specific cis-acting element features. Group I (*ClSK11/12/13*) harbors the most diverse cis-elements and a high abundance of light-responsive elements (Figure 4, Table S5), endowing this subgroup with robust stress adaptation capacity and potential involvement in light-dependent processes. Consistently, *ClSK12/13* are predominantly expressed in floral organs, while *ClSK11* shows high expression in both flowers and roots (Figure 5, Table S6), suggesting their coordinated roles in pollen maturation, sex determination, and basal energy metabolism. Group II (*ClSK21/22/23*) exhibits a high proportion of abundant hormone-responsive elements and photoresponsive elements (Figure 4, Table S5), which may underpin their function in light-dependent developmental processes. *ClSK21/22* are highly expressed in the pericarp and tendrils (Figure 5, Table S6), with their auxin-responsive AuxRR-core and light-responsive elements reflecting a conserved role in modulating plant architecture and longitudinal growth analogous to *OsSK21/22* in rice [50,51,52], while *ClSK23* is upregulated during mid-fruit development (Figure 5, Table S6), coinciding with maximal cell expansion and sucrose import, implying involvement in fruit development and sugar transport. Group III (*ClSK31*) features equal proportions of photoresponsive and hormone-responsive elements with fewer total element types and no complex stress-responsive motifs (Figure 4, Table S5), suggesting specialization in regulating specific tissues or developmental stages under particular photoperiods; its floral-restricted expression (Figure 5, Table S6) further supports potential roles in pollen development or sex determination, alongside auxin, gibberellin, and ethylene response elements. Group IV (*ClSK41*) has the highest proportion of hormone-responsive elements and unique cold-responsive LTR elements (Figure 4, Table S5), indicating a key role in mediating low-temperature adaptation. Notably, *ClSK41* exhibits pronounced fruit-specific expression, especially in ripe, sugar-rich pulp (Figure 5, Table S6), paralleling known carbohydrate regulators such as *ClVST2*, *ClSWEET3*, and *ClAGA2* [48,53], which suggests that *GSK3*-mediated phosphorylation contributes to terminal sugar loading in watermelon fruits. The unique LTR element further facilitates fruit ripening and sugar accumulation under low temperatures. The validation of predicted interactions identified 53 potential interacting partners with five ClGSK3 proteins; excluding unannotated hits, 21 of the 29 functionally annotated interactors are linked to hormone pathways (Table S9). Among these, ClSK21 interacts with the watermelon orthologs of BR-signaling transcription factor BZR1 (Figure 6b and Figure S4), recapitulating the canonical BIN2/AtSK21 interactions characterized in *Arabidopsis* [54,55,56]. Notably, ClSK21 interacts with the BKI1 ortholog (Figure 6b and Figure S4), a conserved component of BR signaling, which differs from the previously reported signaling networks in *Arabidopsis* and rice [54,55,56]. This finding highlights the unique characteristics of BR signaling in watermelon and offers a significant advancement for future research, providing a new entry point for exploring BR-mediated regulatory pathways in this crop. qRT-PCR analysis demonstrates that *ClSK21/31/41* transcripts shift significantly following BL treatment (Figure 7), confirming the involvement of these kinases in BR signaling [16,21,54]. Given that BR promotes fruit enlargement, sugar allocation, stem elongation, and stress tolerance [57,58], the cloned *ClGSK3s*—particularly the BR-responsive triad—provide valuable gene targets for marker-assisted selection aimed at optimizing plant architecture and enhancing soluble solids in watermelon lines.

Promoter dissection, tissue profiles, and validation of predicted interactions of the ClGSK3 family converge to implicate the potential roles in stress tolerance. To systematically dissect the abiotic stress roles of the *ClGSK3s*, we re-mined the published RNA-seq dataset (SRP143549) (Figure S2), profiling four-leaf-stage seedlings of drought-tolerant M20 and drought-susceptible Y34 under 4 d and 8 d water-withholding [59]. The results showed conserved and stage-specific drought response patterns of *ClGSK3s*, such as early up-regulation of *ClSK12/41* and late response of *ClSK11/21/13*. Then we reanalyzed the published RNA-seq dataset (GSE146087) (Figure S3) obtained from six-week-old seedlings of the Crimson Sweet variety exposed to 300 mM NaCl [39], revealing differential expression of *ClGSK3s* under salt stress. This finding is consistent with the established functions of GSK3 proteins in other plant species (e.g., *AtSK11/12* in *Arabidopsis* for drought tolerance [60], *AtSK31* for salt tolerance [23], and *OsSK21* in rice for multi-stress tolerance [24,25]). Evolutionary conservation suggests that *ClGSK3s* may play analogous roles in stress response. The interaction of ClSK21 with BR signaling components further substantiates its involvement in stress tolerance, as BR signaling enhances resistance to stress through the regulation of stress-responsive genes; for instance, rice *GSK3*-regulated BR signaling promotes submergence tolerance [61]. The expression of *ClSK21* and *ClSK22* in pericarp and tendril tissues, which serve as the first line of stress defense, reinforces this potential.

To enhance the understanding of the functional mechanisms of *ClGSK3s* in watermelon and to broaden their applications in breeding, the following targeted recommendations are proposed: First, verify the specific roles of key genes (*ClSK21*, *ClSK31*, *ClSK41*, etc.) in BR signaling, fruit development, and stress tolerance through CRISPR-Cas9 knockout/overexpression techniques, along with phenotypic and physiological analyses of transgenic plants. Second, identify phosphorylation substrates of core proteins, particularly ClSK21, utilizing immunoprecipitation-mass spectrometry (IP-MS) and construct downstream signaling networks via transcriptomic and metabolomic analyses. Third, investigate the response patterns of *ClGSK3s* to multiple hormones, such as auxin and jasmonic acid, and elucidate their crosstalk mechanisms to clarify the integration of hormone signals. Fourth, evaluate the breeding potential of these genes through field trials aimed at developing varieties with optimized plant architecture, improved fruit quality, and enhanced stress resistance using genetic engineering or marker-assisted selection. Finally, explore the regulatory roles of *ClGSK3s* under combined stresses to facilitate the breeding of climate-resilient varieties.

## 4. Materials and Methods

### 4.1. Genome-Wide Identification of GSK3 Genes in Watermelon

To identify *GSK3* family genes in watermelon, we employed the protein sequences of *GSK3s* from *Arabidopsis* and rice as queries to search the watermelon reference genome (version: 97103_v2 Genome) protein database on NCBI (https://www.ncbi.nlm.nih.gov/) using BLASTp with specific criteria: E-value ≤ 1 × 10^−10^ and identity ≥ 40% (accessed on 3 November 2025). Subsequently, we utilized the kinase domain model Pkinase (PF00069) and the hidden Markov model (HMM) of AtSKs protein kinase from the InterPro database (https://www.ebi.ac.uk/interpro/, accessed on 3 November 2025) to search and confirm watermelon GSK3 family members. The protein sequences obtained from both methods were merged, and the conserved domains were validated using SMART (http://smart.embl-heidelberg.de/, accessed on 3 November 2025) and PFAM (http://pfam.janelia.org/, accessed on 3 November 2025). Candidate genes were selected after removing redundancies, and following the nomenclature of *Arabidopsis*, we standardized the names of the identified watermelon GSK3s based on their respective subfamilies and position on the phylogenetic tree. The sequence alignment was generated utilizing the clustalw platform (https://www.genome.jp/tools-bin/clustalw, accessed on 5 November 2025) and enhanced with espript 3 (https://espript.ibcp.fr/ESPript/ESPript/, accessed on 5 November 2025).

### 4.2. Chromosomal Localization and Collinearity Analysis

The chromosomal localization of *GSK3* genes was analyzed by retrieving their loci information from the annotation gff3 files (version: 97103_v2 Genome). The chromosomal positions of *GSK3* genes and their collinearity across watermelon, *Arabidopsis*, and rice were visualized through the functions ‘Gene Location Visualize’ and ‘Dual Synteny Plot for MCScanX’ with default parameters in TBtools (v2.322, Chen C et al., China) [62].

### 4.3. Phylogenetic Tree Analysis

Phylogenetic trees were constructed using IQ-TREE software (v2.4.0, Minh BQ et al., International collaboration (led by researchers from Denmark, Germany, Australia, etc.)) with maximum likelihood (ML) [63]. Sequences of GSK3 family proteins were retrieved from previous studies [10,42,43,44,45,46,47] and aligned using the MAFFT online tool (https://www.ebi.ac.uk/Tools/msa/mafft/, accessed on 12 January 2026) with default parameters. The aligned sequences were utilized as input with the following settings: Q.plant+G4 model, 1000 bootstrap replicates, and 1000 SH-aLRT tests for branch support. The phylogenetic tree was enhanced using the online tool iTOL (https://itol.embl.de/, accessed on 12 January 2026).

### 4.4. Expression Profiles of ClGSK3 Genes

Transcriptome data from previous investigations were obtained from the NCBI database. The raw sequencing reads were processed to obtain high-quality clean reads, which were then re-mapped to the updated watermelon reference genome (97103v2). Subsequently, gene expression levels were normalized (TPM), converted to log2 ratios, and visualized using TBtools software (v2.322, Chen C et al., China).

### 4.5. Cis-Acting Element Analysis of GSK3 Genes in Watermelon

The 2000 bp upstream sequence information of the *GSK3* genes was retrieved from the gff3 annotation file for analysis of cis-acting elements in the gene promoters. An analysis was conducted utilizing the online tool PlantCARE with default parameters (https://bioinformatics.psb.ugent.be/webtools/plantcare/html/, accessed on 19 November 2025), and the outcomes were visualized through the ‘simple biosequence viewer’ function of TBtools (v2.322) with default parameters.

### 4.6. PPI Network Prediction

To identify potential proteins that may interact with GSK3 family proteins in watermelon, we obtained protein–protein interaction data for *Arabidopsis* from the STRING database (https://string-db.org/, accessed on 1 December 2025), applying a medium confidence threshold (Required score > 0.700). A BLASTP search of the watermelon protein database was then conducted using the interaction proteins from *Arabidopsis* via the ‘PPI Prediction’ feature in TBtools. The results were subsequently visualized using Cytoscape software (v3.10.3, Cytoscape Consortium, San Diego, CA, United States).

### 4.7. Y2H Assays

For the GAL4-based Y2H assay, the CDS of ClSK21 and the six high-confidence interacting proteins were cloned into the pGADT7 vector at the EcoRI and BamHI restriction sites through gene synthesis by GenScript Corporation (Nanjing, China). The gene-synthesized fragments were subsequently excised from pGADT7 with EcoRI and BamHI and ligated into the matching sites of pGBKT7 using T4 DNA ligase. The interaction assays were conducted with a Yeast Transformation Kit (YT0010, Beijing LABLEAD Trading Co., Ltd., Beijing, China) following the manufacturer’s instructions. The DMSO (ID9011) used in the experiment was purchased from Beijing Solarbio Science & Technology Co., Ltd. (Beijing, China). Both the bait and prey constructs were co-transformed into the yeast strain AH109 and cultured on SD/-Leu/-Trp medium, followed by selection on SD/-Leu/-Trp/-His/-Ade medium.

### 4.8. Quantitative Real-Time PCR

The homozygous inbred line TP101 was used in the exogenous BR treatment experiment, which was established by the Institute of Cash Crops, Hebei Academy of Agriculture and Forestry Sciences. The exogenous BL treatment experiment was performed as described previously [5]. The 3-day-old hydroponically grown seedlings were treated with 1 µM exogenous BR or a solvent-only control (0 µM BR). After 4 days of treatment, total RNA was isolated from all seedlings of both treated and control groups (three biological replicates each) using Trizol reagent (15596026CN, Invitrogen, Carlsbad, CA, USA), and the quality was checked by agarose (CA1341, Beijing Coolaber Science & Technology Co., Ltd., Beijing, China) gel electrophoresis. Subsequently, cDNA was generated with a QuantiTect Reverse Transcription Kit (JKR23014, GeneCreate, Wuhan, China) and employed as a template for quantitative real-time PCR (qRT-PCR) analysis. qRT-PCR assays were conducted on an Applied Biosystems 7500 real-time PCR system, employing the 2 × SYBR Green qPCR Mix (AH0105-B, Shandong Sparkjade Biotechnology Co., Ltd., Jinan, China), following the manufacturer’s recommended protocol. Each trial was replicated three times using distinct biological samples. Expression levels were normalized to the internal control gene *ClCAT* [64] using the 2^(−ΔΔCt)^ method, while all quantitative primers were listed in Table S10.

## 5. Conclusions

This study presents the first comprehensive identification and characterization of the watermelon *GSK3* gene family, which consists of eight evolutionarily conserved members. *ClGSK3s* integrate BR and other hormone signaling pathways to regulate watermelon fruit development, plant architecture, and abiotic stress tolerance. Notably, ClSK21 serves as a key regulator of BR signaling through its interactions with BKI1/BZR1 orthologs. These findings enhance the understanding of the diversity within the plant *GSK3* family and provide prioritized molecular targets for breeding high-quality, stress-resistant watermelon varieties, thereby establishing a foundation for further functional research and genetic improvement.

## Data Availability

The original contributions presented in this study are included in the article/Supplementary Material. Further inquiries can be directed to the corresponding author.

## Supplementary Materials

The following supporting information can be downloaded at https://www.mdpi.com/article/10.3390/plants15030484/s1. Figure S1. Multiple sequence alignment of the ClGSK3 protein family. Figure S2. The expression profile of *ClGSK3s* genes under drought-tolerant conditions. Figure S3. The expression profile of *ClGSK3s* genes under salt stress. Figure S4. GAL4-based Y2H assay validation of the interaction of ClSK21 and 6 predicted interaction proteins. Table S1. Physicochemical parameters of 8 *GSK3* genes in *C. lanatus* genome. Table S2. Physicochemical parameters of *GSK3* genes in *Arabidopsis* and rice. Table S3. The collinear pairs of *GSK3* genes among watermelon, *Arabidopsis* and rice. Table S4. The orthogroups of collinear pairs of *GSK3* genes in watermelon, rice and *Arabidopsis*. Table S5. The cis-regulatory elements predicted for *GSK3s* genes in *C. lanatus*. Table S6. The expression levels of *ClGSK3* genes in different tissues of watermelon. Table S7. The expression levels of *ClGSK3* genes under drought-tolerant conditions. Table S8. The expression levels of *ClGSK3* genes under salt stress. Table S9. Prediction analysis of the interacting proteins of the watermelon *GSK3* gene family. Table S10. Primer sequences used in this study.
