# Supplementary material for: Genome-Wide Identification, Characterization, Expression Analysis, and Interacting Protein Prediction of the GSK3/Shaggy-like Gene Family in Watermelon
Source: Plants (Basel). 2026 Feb 4;15(3):484. doi: 10.3390/plants15030484 (PMC12899405; doi:10.3390/plants15030484)
Supplement: Supplementary file 1 [file plants-15-00484-s001.zip › Additional Figures index.pdf]

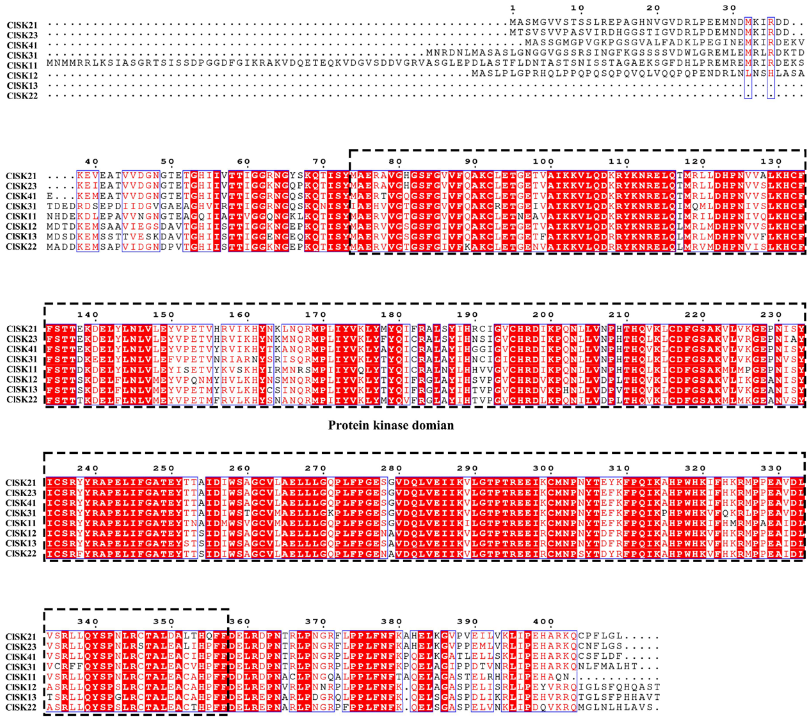

Figure S1. Multiple sequence alignment of the ClGSK3 protein family. The 8 ClGSK3 paralogs were aligned based on their two conserved domains: Protein kinase domain was highlighted and delineated by dashed lines. The region enclosed in blue box signifies a predominantly conservative or highly similar fragment. Fully conserved sequences are shaded in red, while similar sequences are denoted in red font. Gaps are represented by dots. Sequence alignment diagrams were generated utilizing the clustalw platform (https://www.genome.jp/tools-bin/clustalw) and enhanced with espript 3 (https://espript.ibcp.fr/ESPript/ESPript/).

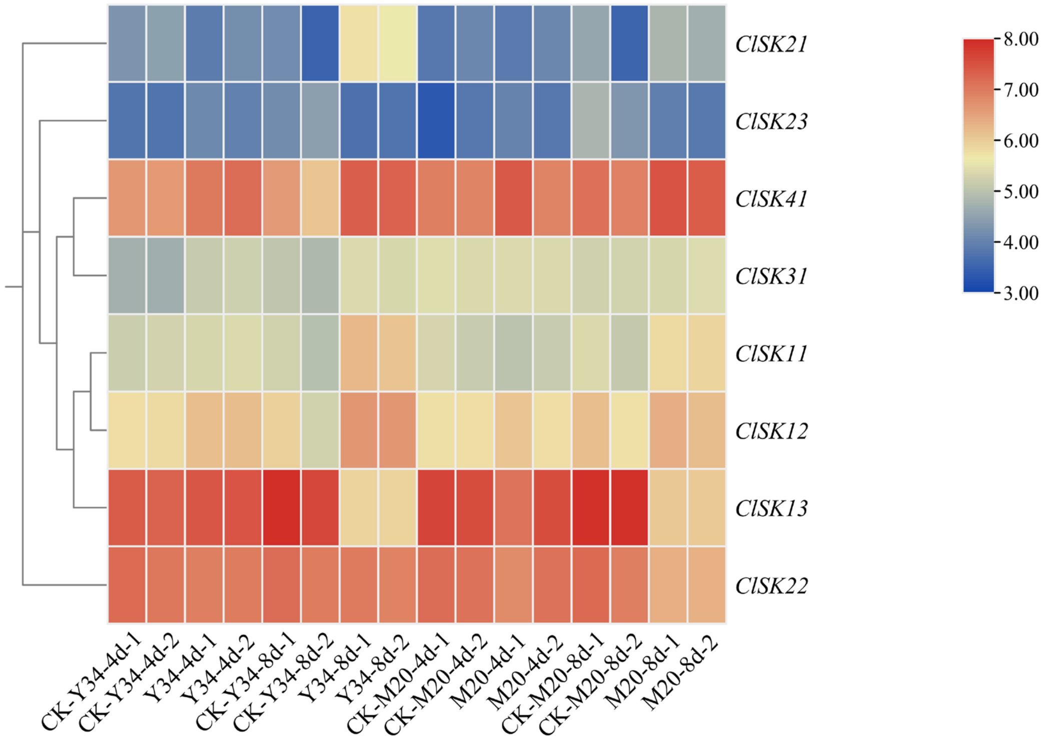

Figure S2. The expression profile of *ClGSK3s* genes under drought-tolerant conditions. Four-leaf-stage seedlings of drought-susceptible Y34 and drought-tolerant M20 were subjected to water withholding for 4 d and 8 d; fully irrigated plants (CK) served as controls. The color scale indicates fold changes normalized by log2 transformed data, with blue signifying downregulated genes and red indicating upregulated genes.

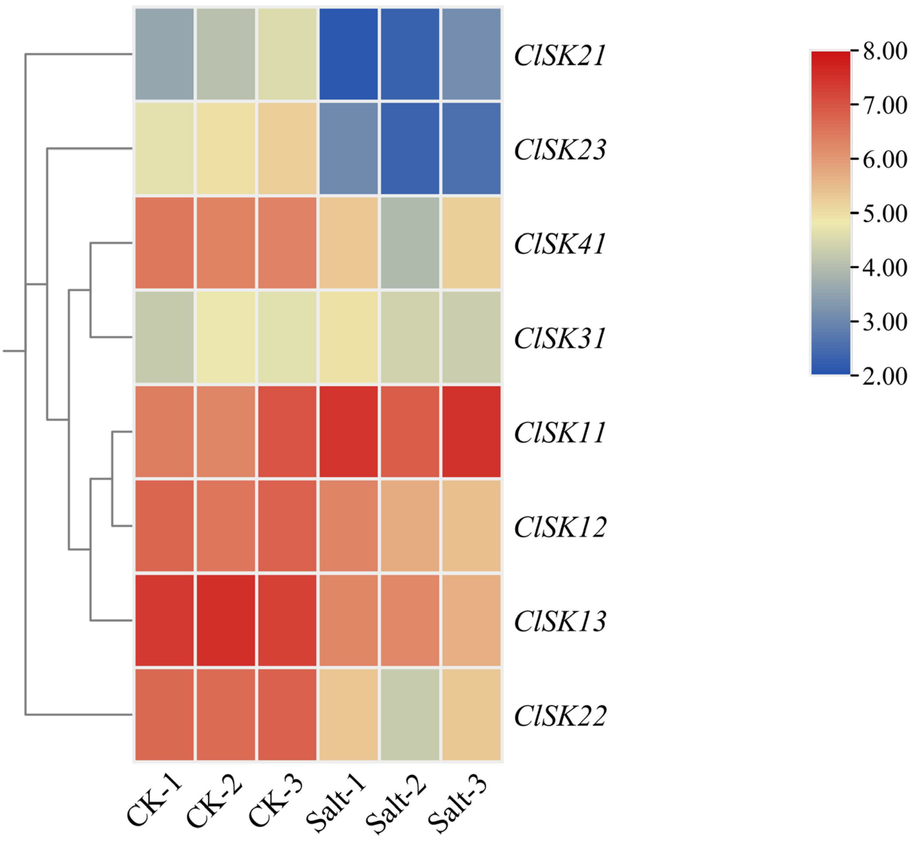

Figure S3. The expression profile of *ClGSK3s* genes under salt stress. Six-week-old seedlings of the Crimson Sweet variety exposed to 300 mM NaCl; deionized-water-treated plants (CK) served as controls. The color scale indicates fold changes normalized by log2 transformed data, with blue signifying downregulated genes and red indicating upregulated genes.

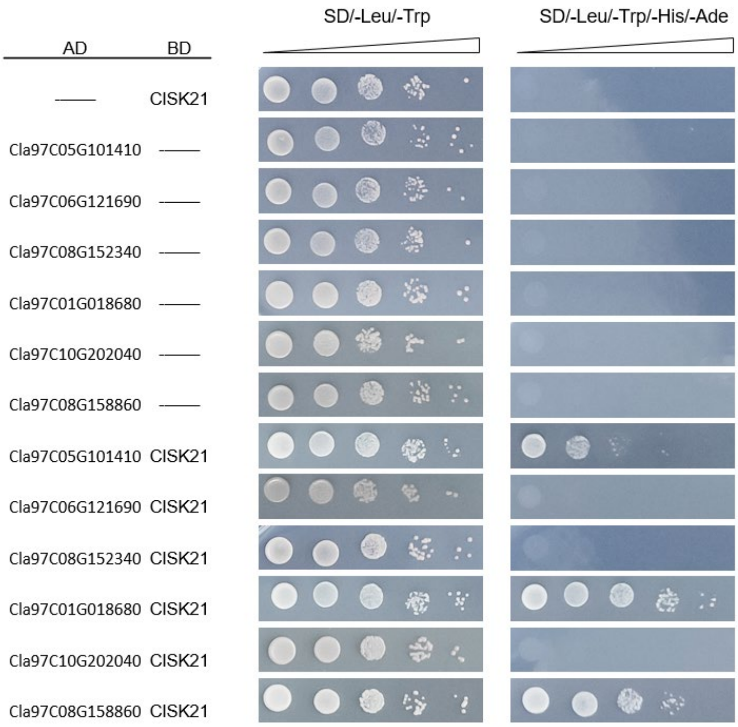

Figure S4. GAL4-based Y2H assay validation of the interaction of ClSK21 and 6 predicted interaction proteins. AD, activation domain; BD, binding domain; SD, synthetic dropout; gradients indicate tenfold serial dilutions.
